# Feasibility of self-organized blood sample collection in adults for study purposes in a primary care setting

**DOI:** 10.1371/journal.pone.0286014

**Published:** 2023-05-25

**Authors:** Dominik Schröder, Frank Müller, Gloria Heesen, Eva Hummers, Alexandra Dopfer-Jablonka, Kai Vahldiek, Frank Klawonn, Sandra Steffens, Marie Mikuteit, Jacqueline Niewolik, Stephanie Heinemann

**Affiliations:** 1 Department of General Practice, University Medical Center, Göttingen, Germany; 2 Department of Rheumatology and Immunology, Hannover Medical School, Hannover, Germany; 3 German Center for Infection Research (DZIF), Partner Site Hannover-Braunschweig, Germany; 4 Department of Computer Science, Ostfalia University of Applied Sciences, Wolfenbuettel, Germany; 5 Biostatistics Group, Helmholtz Centre for Infection Research, Braunschweig, Germany; Universidad Nacional Hermilio Valdizan Escuela Academico Profesional de Medicina Humana, PERU

## Abstract

**Background/aims:**

The COVID-19 pandemic situation poses new challenges for research. Ethical issues might arise if especially vulnerable individuals for severe COVID-19 course expose themselves because of participation in studies to a higher risk of infection for study purposes. How is the feasibility and acceptance of self-organized blood sample collections to measure anti-SARS-CoV-2 Spike IgG antibodies in persons with a high risk for a severe COVID-19 disease progression?

**Methods:**

Persons with a high risk for a severe COVID-19 disease progression (immunocompromised, oncology patients or over 80 years old) were recruited between January and September 2021 to send in blood samples (at least 500 μl) 1 month and 6 months after second COVID-19 vaccination. Participants were given the choice of drawing capillary or venous blood themselves or having blood drawn by health professionals belonging to either the study’s own research team or the personnel found in local practices or clinics. Participants were surveyed via a telephone interview in December 2021 and January 2022 about their choice of blood sampling methods and influence of blood collection choice upon study participation.

**Results:**

Data from 360 participants was collected via telephone follow-up. First blood samples were collected by the participants themselves (35.8%), local practices or clinics (31.9%) and the research team (22.5%). Second blood samples were mostly collected in local practices or clinics (35.6%) followed by participants themselves (25.9%) and the research team (11.5%). Blood samples were not collected in 2.5% and 19.1% of persons during first and second blood draw, respectively. Only 2% of blood samples did not reach the laboratory or were not analyzable. About one-fourth (26%) of participants stated that they would not have participated in the study if it would have been required to travel to the university hospital to give their blood sample.

**Conclusions:**

Participants were able to self-organize blood collection, making use of several different blood sample methods. Nearly all blood samples were analyzable when self-collected and sent in by post. One-fourth of the participants would not have participated in the study if required to give their blood sample in the study location.

**Trial registration:**

German Clinical Trial Registry, DRKS00021152.

## Background

The COVID-19 pandemic caused by SARS-CoV-2 affects daily life in various ways due to regional lockdowns and other measures to minimize the spread of the virus [1–5]. Various factors can lead to an increased risk for severe COVID-19 risk and death such as advanced age and various pre-existing conditions, e.g. cancer and autoimmune diseases and many other chronic diseases [6–8]. Especially these vulnerable groups are of high research interest. This includes research on the pathological processes caused by SARS-CoV-2 and COVID-19, but e.g. by COVID-19 vaccination [9–13].

Research on these vulnerable groups using conventional methods like blood sampling in the research facility may expose the individuals to an additional infection risk e.g. due to transportation and increased personal contact. This is especially concerning when study related bloodwork is adhering to another schedule than the regular appointments were standard-care blood analysis is performed. The pandemic situation has highlighted the researchers’ responsibility to reduce infection risk especially in vulnerable populations [14]. Contrary, the option to reduce contact may also increase participation in the study and may reach participants e.g. in remote areas that would be otherwise unlikely to participate in studies. Hall et al. (2020) evaluated the willingness to use home collection methods to provide specimens for COVID-19 research. Participants were more willing to use at home collection compared to collection methods in clinics [15]. In Diabetes type 2 patients, a home capillary blood collection kit showed high patient satisfaction during the pandemic with comparable serological results to clinic-based venous blood collection [11]. Also in SARS-CoV-2 serological research, the usage of capillary blood is comparable to venous blood which opens up a new methods for blood self-collection in clinical trials and virtual care to patients [16, 17]. Capillary blood for studies has up until now mostly been collected from the fingertip using safety lancets [18]. In the future, capillary blood may also be collected from the upper arm using inventions like the TASSO device [17].

This study aims to evaluate the feasibility of self-organized blood sample collection for SARS-CoV-2 antibody screening in persons with a high risk for a severe COVID-19 disease progression. Following hypothesis will be examined:

- Participants use various options to collect blood samples if available
- Blood sample of participants are analyzable for SARS-CoV-2 antibodies no matter which blood sample option was used
- The choice of self-capillary blood draw is associated with sociodemographic factors
- Less participants would participate in the study if they had to get blood drawn only by study personnel

## Methods

### Research design and participants

The Covid-Contact Immune Study (CoCo Immune Study) is a longitudinal, prospective, observational study and was conducted at two large Hospitals in Göttingen and Hannover in Germany [19]. Beginning in March 2021, we recruited persons who (1) were 18 years or older, (2) capable of giving consent and (3) persons with a high risk for a severe COVID-19 course (80+ years old, hematological or oncological disease or immunosuppressed participants) and intended to get the COVID-19 vaccination. Exclusion criteria were (1) refusal to provide informed consent or (2) contraindications to capillary blood collection (hematophobia, chronic wounds at puncture site). There were no further inclusion or exclusion criteria. Detailed information about the inclusion and exclusion criteria can be found in the study protocol [19]. The last participant was recruited in September 2021.

Using newspaper advertisements and informational posters in hospitals, vaccination centers and in rheumatological doctors´ offices, we recruited participants. Due to the vulnerability of our target group, we organized the study in a minimized-contact manner. Interested people could contact the study site by calling the study phone or writing an e-mail to the study mail address. Therefore, the sample is a convenience sample. The enrollment in the study and the declaration of consent by the participants could be given by telephone or videocall or in person. For any persons wishing to be enrolled on the phone or during a videocall, we shipped the study material in advance per post to the participants’ homes. The study team encouraged every participant to take all possible preventive measures to avoid becoming infected by the SARS-COV-2 virus and to observe all national, state and local pandemic regulations. In the case of enrollment via telephone or videocall, the signed consent form was returned by mail.

In December 2021 and January 2022, participants were contacted in a short telephone follow-up interview to gain additional information about a possible third COVID-19 vaccination, pausing therapy before or after vaccination and the participants’ experiences with self-organized blood draws. Further information about the CoCo Immune Study can be gathered in the study protocol [19].

### Blood sample collection

Blood samples were retrieved within the CoCo Immune study where participants were asked to collect at least 500 μl blood at two time points using capillary blood or venous blood. The study participants were free to decide where and how they would like to have their blood drawn. A telephone hotline was set up where participants could make an appointment at the university medical center for venous blood draw through a healthcare professional. No method of blood collection was recommended by the study team. Blood collection sets including sample tubes, sterile automatic lancing devices, bandage patches, aseptic swaps, alcoholic pads to disinfect the incision site and an illustrated leaflet describing the capillary blood take were given to every participant along with shipping return boxes at study enrollment. Safety lancets of SARSTEDT (Sarstedt AG & Co. KG, Nümbrecht, Germany) with an insertion depth between 1.6mm and 1.8mm with a thickness between 1.2mm and 1.5mm were provided for capillary blood collection at home (S1 Fig in S1 File). In addition, 500 μl blood collection tubes (SARSTEDT Microvette^®^ 500 K2 EDTA) were provided for each blood draw (S1 Fig in S1 File). Each study participant received instructions about the use of the safety lancets at enrollment and was provided with further education as needed. The first blood sample was due 30 days and the second blood sample was due 6 months (180 days) after the second COVID-19 vaccination. Participants shipped the self-collected blood samples to the research lab of the Hannover Medical School using prepaid shipping boxes provided by the study team at enrollment. In these shipping boxes, participants placed both their blood samples as well as the corresponding questionnaires, which were given out at study enrollment. The blood samples were screened with a quantitative ELISA for anti-SARS-CoV-2 spike protein 1 (S1) immunoglobulin G (IgG). The CE Certified version of the Anti-SARS-CoV-2-QuantiVac-ELISA from Euroimmun, Lübeck, Germany, was used. Participants did not incur any costs for the laboratory tests or provided study material and received no compensation for their participation.

### Measured variables

Sociodemographic variables were collected in the baseline questionnaire including sex, age, school education, city resident size, comorbidities, if they work in a nursing or medical profession and COVID-19 risk group. Participants in the COVID-19 risk group *Immunosuppressed* were further divided into the groups conventional immunosuppressants (e.g. Methotrexate, Azathioprine), corticoids (e.g. Prednisone, Hydrocortisone), TNF inhibitor (e.g. Etanercept, Infliximab), other biologics (e.g. Tocilizumab Ustekinumab) and others. School education was categorized as low, middle, reflecting the German secondary school education. City resident size was included because larger cities have higher physician density and therefore more opportunities for blood collection by health care specialist. Additionally, at baseline, participants stated their experience with injections and blood sugar measurements measured with a five-point Likert scale where higher values indicate less experience and their intended blood draw method at study enrollment.

In a computer-assisted telephone follow-up interview, the participants were asked several questions about the blood collection methods and the feasibility of self-organized blood collection. For example, participants were asked which blood draw method they used for their first and second blood draw. Categories for blood-draw methods included self-capillary blood-collection, in a local practice or clinic, by study personnel or other methods (e.g. venous-blood draw by a family member working as nurse or at work in a clinic). Medical provider in practices or clinics might consider additional blood collection as an out-of-pocket service since it is for study purposes and not related to standard care. Therefore, we asked the participants if they had to pay for their blood draw in practices or clinics. Additionally, participants were asked if they would still have participated in this study even if they would have had to get blood drawn in the institutes of the study (either University Medical Center Göttingen or Hannover Medical School).

### Ethics approval and consent to participate

The study was conducted according to the guidelines of the Declaration of Helsinki and approved by the Institutional Review Board of Hannover Medical School (9948_BO_K_2021) and University Medical Center Göttingen (29/3/21). All participants provide written consent to participate in this study. The study is registered in the German register for clinical trials (DRKS00021152). Participants did not incur any costs for the laboratory tests or provided study material. Participants also did not receive financial compensation.

### Statistical analysis

Participants were included in the statical analysis if they were reached during the telephone follow-up. Participants who did not draw their second blood sample during the time of the follow-up interview were excluded from the data analysis. Also, patients who had not yet had 180 days after the second vaccination were excluded from the analysis about the second blood draw. Patients’ characteristics were described with the absolute number and proportion. Additionally, age was reported with the mean and standard deviation. McNemar’s test was used to test for change in participants that intended to draw capillary blood themselves and participants that did not intend to do so and their actual decision. Univariate and multivariable logistic regression was performed to explore factors associated with self-capillary blood collection at both blood draws including gender, age, school education, city resident size, nursing or medical profession and self-reported experience with injection of drugs or blood sugar measurements. All statistical analysis was done by using R (Version 4.1.2).

## Results

**Fig 1:**
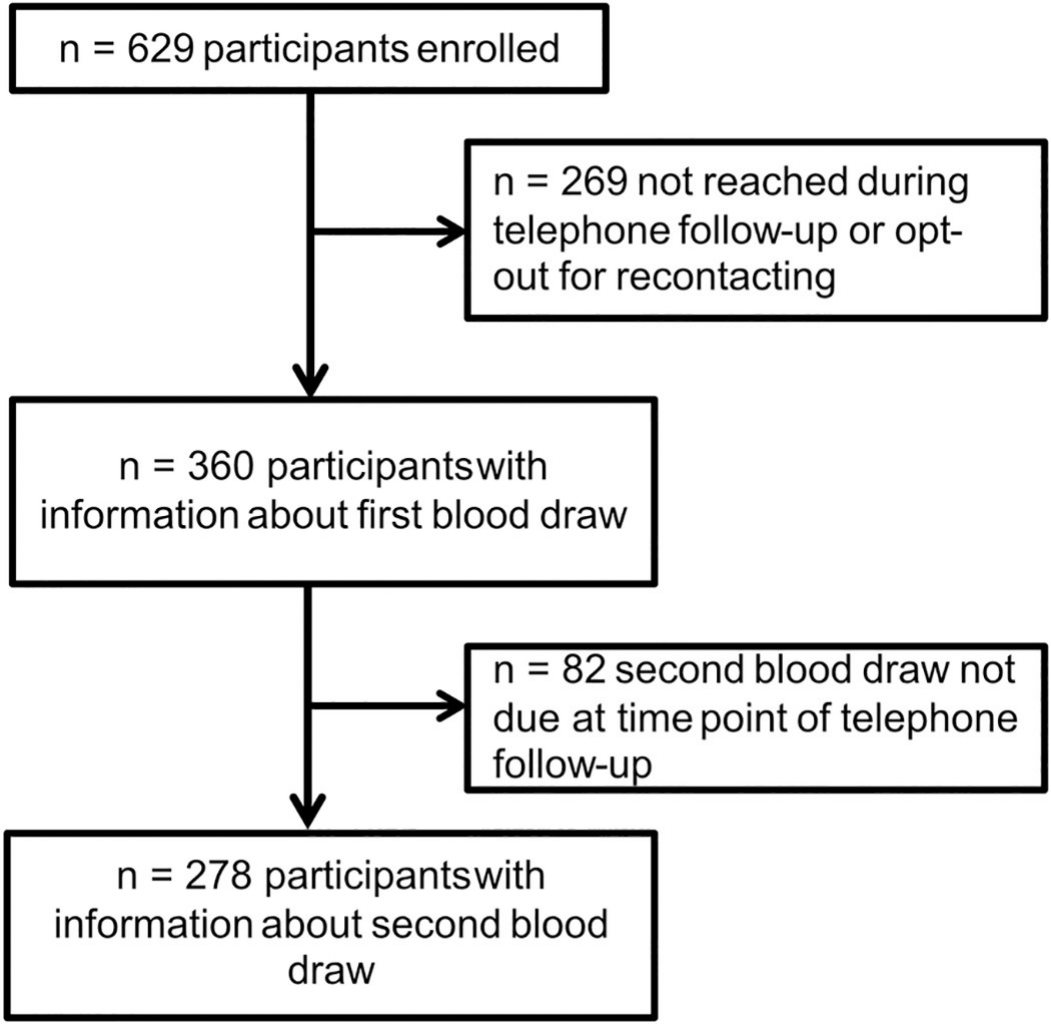
Flowchart of participant enrollment and data analysis.

At start of the study 629 participants were enrolled from which from 360 participants were reached during telephone follow-up (Fig 1). The sociodemographic characteristics of non-responders can be found in the supplement (S1 Table in S1 File). At the time of the second follow-up, the second blood sample was not yet due for 82 participants.

In the analysis, included participants were mostly female (58.5%), immunosuppressed, had a high-level secondary school education (47.8%) and lived in rural areas (44.8%). About one-third of all participants had hypertension (Table 1).

**Table 1:** Participant characteristics (N = 360).

Sex	n (%)
Male	142 (39.4)
Female	200 (55.6)
Missing	18 (5.0)
Age	
Mean (standard deviation)	58.1 (14.8)
< 40 years	46 (12.8)
40–65 years	183 (50.8)
> 65 years	126 (35.0)
Missing	5 (1.4)
High risk group*	
80+ years old	27 (7.5)
Immunosuppressed*	203 (56.4)
Conventional immunosuppressants	89 (43.8)
Corticoids	63 (31.0)
TNF inhibitor	37 (18.2)
Other biologicals	20 (9.9)
Other	51 (14.2)
Haematological or oncological disease	139 (38.6)
School education	
Low	61 (16.9)
Middle	109 (30.3)
High	162 (45.0)
Not specified	7 (1.9)
Missing	21 (5.8)
City resident size	
< 5,000	152 (42.2)
5,000–20,000	68 (18.9)
20,000–100,000	45 (12.5)
> 100,000	74 (20.6)
Missing	21 (5.8)
Nursing or medical profession	
yes	30 (8.3)
Co-Morbidities*	
Hypertension	132 (36.7)
Heart failure	9 (2.5)
Diabetes type 1	4 (1.1)
Diabetes type 2	24 (6.7)
Chronic obstructive pulmonary disease	7 (1.9)
Missing	9 (2.5)

Data is n (%) if not otherwise stated;

*multiple selection possible

**Fig 2:**
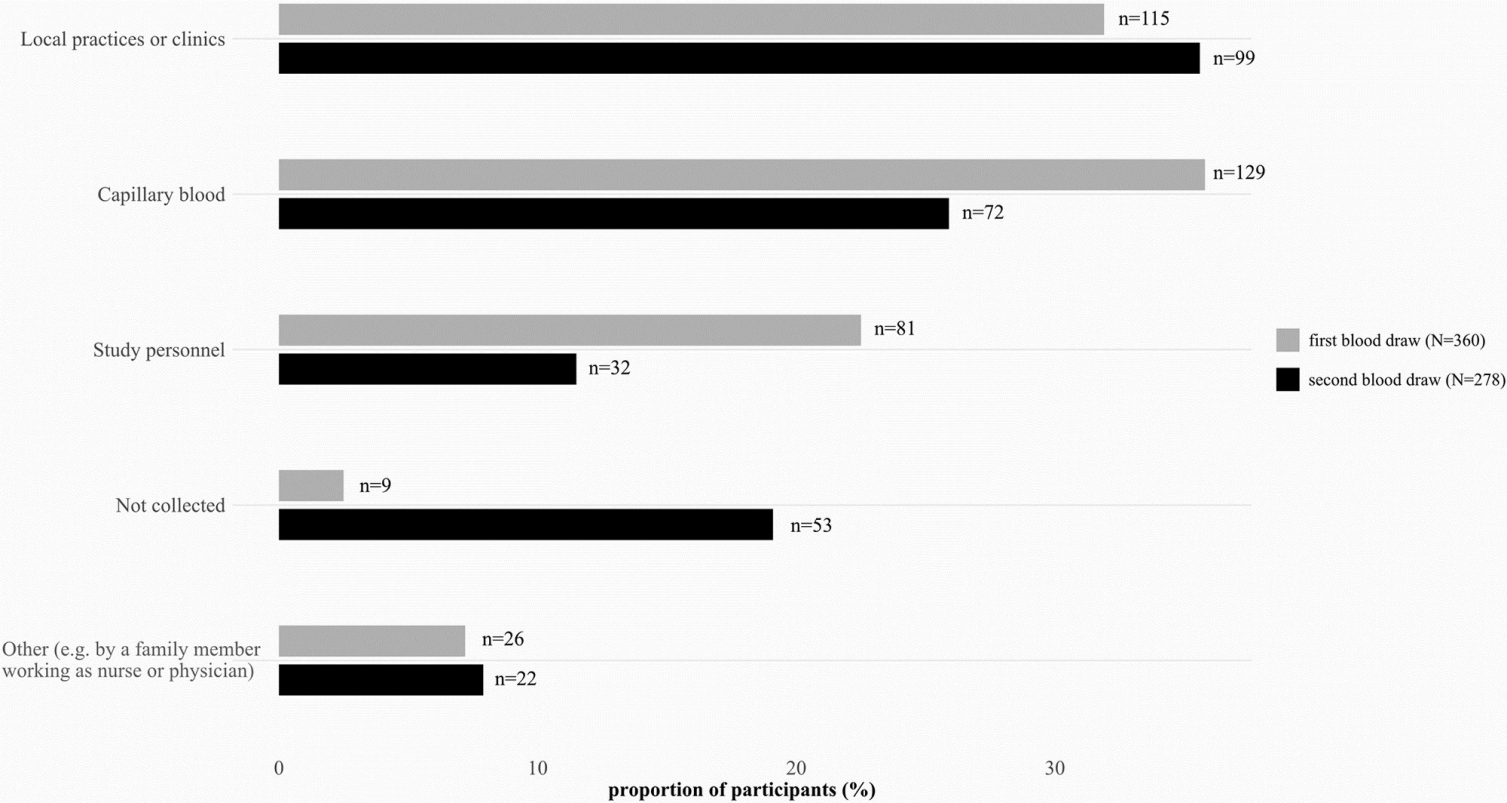
Used blood collection method in first (N = 360) and second blood draw (N = 278).

For the first blood draw, most participants (35.8%) collected capillary blood themselves, which decreased during second blood draw (25.9%) (Fig 2). Gathering blood samples in local practices or clinics was used mostly during the second blood draw timepoint (35.6%). Getting blood drawn by study personnel in the research facility decreased from 22.5% in the first blood draw to 11.5% in the second blood draw. The rate of participants who reported that they draw blood successfully but their sample was eventually not analyzed in the lab increased from 2.5% for the first blood draw to 19.1% for the second blood draw.

About 78.7% of participants who collected their blood twice did not change their blood draw method between the first and second blood draw. The changes between the blood collection method can be seen in Table 2 outside of the diagonal cells (Table 2). In participants that collected their blood in both follow-ups the highest relative change in blood collection methods was observed in participants gave blood by the study personnel (33.3%), followed by self-capillary blood draw (26.4%), other (e.g. venous-blood draw by a family member working as nurse or at work in a clinic) (15.8%) and local practices or clinics (11.3%). One of 128 participants (0.7%) who had blood drawn in local practices or clinics paid for the blood draw as an out-of-pocket expense.

**Table 2:** Changes in blood draw method in participants with two blood draws (N = 278).

**Second blood collection**							
**First blood collection**		Self- capillary	Local practices or clinics	Study personnel	Other	Not collected	**All**
	Self capillary	64 (61.5)	18 (17.3)	3 (2.9)	2 (1.9)	17 (16.4)	104 (100)
	Local practices or clinics	4 (4.0)	71 (71.0)	3 (3.0)	2 (2.0)	20 (20.0)	100 (100)
	Study personnel	3 (4.2)	8 (15.7)	26 (50.1)	2 (3.9)	12 (23.5)	51 (100)
	Other	1 (4.3)	2 (8.7)	0	16 (69.6)	4 (17.4)	23 (100)
	Not collected	0	0	0	0	0	0 (100)

Data is n (% by row)

### Self-capillary blood collection

Only 51.7% and 44.1% of the study participants who intended at the beginning of the study to draw blood themselves using the capillary blood collection kit, reported they did as intend at first and second blood draw, respectively (Tables 3 and 4). The McNemar-Test shows a significant change between the intended self-capillary blood collection and the actual blood collection method during the first (p <0.001) and second blood collection (p <0.001).

**Table 3:** Intended and carried out self-capillary blood collection during first blood collection (N = 309).

	First blood collection			
intended		yes	no	
	yes	107 (51.7)	100 (48.3)	207 (100)
	no	12 (11.8)	90 (88.2)	102 (100)

Data is n (% by row); p <0.001 using McNemar’s-Test

**Table 4:** Intended and carried out self-capillary blood collection during second blood collection (N = 203).

	second blood collection			
Intended		yes	no	
	yes	63 (44.1)	80 (55.9)	143 (100)
	no	3 (5.0)	57 (95.0)	60 (100)

Data is n (% by row); p <0.001 using McNemar’s-Test

When exploring influencing factors in persons that draw capillary blood themselves at both time points city resident size, age and the experience with injection of drugs or blood sugar measurements were significantly associated (Table 5). Participants in small cities (<5,000 residents) were less likely (OR = 0.42 (95% CI: 0.41; 0.96), p = 0.03) and younger participants (<40 years) were more likely (OR = 3.12 (95% CI: 1.20; 8.15), p = 0.02) to draw blood themselves using capillary blood compared to cities with more than 100,000 participants and participants older than 65 years, respectively. Participants reporting a higher experience with injection of drugs or blood sugar measurements were more likely to draw capillary blood themselves (OR = 0.83 (95% CI: 0.70; 0.99), p = 0.04). Gender, school education, a nursing or medical profession were not significantly associated with self-capillary blood draw. When using a multivariate model only city resident size remained a significant predictor of self-capillary blood draw. Adjusted for all other predictors residents in cities with less than 5,000 residents were significantly more less likely to draw capillary blood themselves compared to participants in cities with more than 100,000 residents (OR = 0.42 (95% CI: 0.19; 0.92), p = 0.04).

**Table 5:** Logistic regression predicting carried out self-capillary blood draw at both time points.

Variable	Univariate OR (95% CI)	p	Multivariate OR (95% CI)	p
Gender				
Male	0.61 (0.31; 1.20)	0.15	0.67 (0.32; 1.39)	0.28
Female	- (ref)	-	- (ref)	-
School education				
Low	0.51 (0.19; 1.39)	0.19	0.81 (0.26; 2.49)	0.71
Medium	0.72 (0.37; 1.41)	0.34	1.07 (0.51; 2.27)	0.86
High	- (ref)	-	- (ref)	-
City resident size				
<5,000	0.42 (0.19; 0.92)	0.03	0.41 (0.17; 0.96)	0.04
5,000–20,000	0.50 (0.20; 1.30)	0.16	0.39 (0.13; 1.15)	0.09
20,000–100,000	0.49 (0.16; 1.55)	0.23	0.39 (0.12; 1.33)	0.13
>100,000	- (ref)	-		
Age				
< 40 years	3.12 (1.20; 8.15)	0.02	2.75 (0.91; 8.35)	0.07
40–65 years	1.11 (0.55; 2.23)	0.78	1.03 (0.46; 2.31)	0.94
> 65 years	- (ref)	-	- (ref)	-
Nursing or medical profession				
Yes	1.45 (0.45; 4.65)	0.53	2.11 (0.60; 7.42)	0.24
No	- (ref)	-	- (ref)	-
Experience with injection of drugs or blood sugar measurements				
Likert-Scale 1–5 (higher values indicate less experience)	0.83 (0.70; 0.99)	0.04	0.83 (0.68; 1.00)	0.051

Of the 351 participants who reported sending in blood at the first time point, 7 (2.0%) blood samples were not received or could not be analyzed.

In the telephone follow-up, 25.2% of the participants stated that they would not have participated in the study had they been required to travel to the university hospital to draw blood.

## Discussion

The aim of this study is to evaluate the feasibility of self-organized blood sample collection for SARS-CoV-2 antibody screening in persons with a high risk for a severe COVID-19 disease progression. Study participants were asked to self-organize blood draws one and six months, respectively, after second COVID-19 vaccination and send the blood samples per mail to the research institute. Blood collection tubes and safety lancets were given to every participant along with a prepaid, addressed shipping boxes at study enrollment. The most common blood collection methods were *self-collected capillary blood* and *blood draws in local practices or clinics*. Blood was not collected in 2.5% and 19.1% of the study population one month, respectively six months, after second COVID-19 vaccination. Participants in small cities were more likely to draw capillary blood themselves at both time points. Nearly all blood samples sent to the study center by mail were received and could be analyzed. About one-fourth of the participants would not take part in this study if they had to draw blood in the university hospital.

Study participants were able to draw their own blood using safety lancets provided by the study personnel. The safety lancets used had a penetration depth of 1.6mm, blade width of 1.5mm and are designed for blood draws up to 500 μl according to the manufacturer. The study participants were requested to draw 500 μl of blood when using safety lancets. Serafin et al. investigated the blood volume collected after a single lancing of the fingertip and after gently pressing the finger for two minutes [20]. The highest mean blood volume of 265 μl had the Prolance Max Flow with a blade width of 1.5mm and 1.6mm penetration depth which is identical to the lancets used in this study. The safety lancet with the highest mean blood volume was associated with the highest pain perception. The high requested blood volume for capillary blood draw in our study of 500 μl and the associated pain perception may explain the reduced proportion of participants collecting capillary blood in the second blood draw compared to the first blood draw. This could also be a reason for the discrepancy between intended and performed capillary blood drawing. Negative experiences of the first blood collection could influence the choice of the second blood collection option or even leading to not collection blood at the second time point. Most studies investigating SARS-CoV-2 antibody response using a longitudinal study design do not report loss-to follow-up [21–24]. The study of Abraha et al. surveyed antibody response to SARS-CoV-2 up to 12 months after infection using conventional venous blood draw performed by phlebotomists. Here, out of 149 enrolled participants, 21 participants were lost at first follow up and 6 participants were lost at second follow-up (total loss-to-follow up rate 14.1%) [25]. In our study we found a comparable loss-to-follow-up rate of 19.1% after second blood draw.

The choice of a blood collection method and their acceptability may differ with different study populations. Our study consisted only of elderly, immunocompromised persons or persons with a hematological/oncology illness. Our results show a lower chance of drawing capillary blood twice in participants living in city with less than 5,000 residents compared to participants living in bigger cities. Other factors like age and school education did not indicate an association with preference of capillary blood draw. Additional sociodemographic and medical variables need to be studied in different patient groups as these may influence the choice of blood draw options. Also the usage of more user-friendly devices for self-administered capillary blood collection like the TASSO-SST device could lead to a higher response rate [17]. Muehlensiepen et al. interviewed rheumatic patients and health care professionals about possible at-home blood self-sampling using an upper-arm device like TASSO-SST to draw blood themselves [26]. Patients reported an easy application and high usability. Patients and health care professionals saw possible cost and time savings and a reduction of risk of infection during COVID-19 as benefits. Further research should focus on combining qualitative and quantitative methods to gain more insight about further influencing factors and motives of the blood draw option choices.

### Limitations

This study comes with several limitations. The results of our study cannot be considered representative for all participants with a high risk for a severe COVID-19 course due to the convenience sampling method and a possible loss-to follow-up bias. Non-responders were older and less educated compared to participants reached in the telephone follow-up. Our data did not provide information about why blood samples were not analyzed. Therefore, we cannot distinguish if blood samples were not received (e.g. package got lost in mail) or if the sample could not get analyzed (e.g. due to insufficient sample volume). Information about the reason for the choice of blood collection method or pain perception during the self-blood collection were not collected. This information should be gathered in further research to gain more detailed information.

## Conclusion

In our study population with persons with a high risk for a severe COVID-19 course, self-organized blood collection was an feasible way to screen for SARS-CoV-2 antibody. Participants mostly drew capillary blood themselves or had venous blood drawn in local practices or clinics. About one-fourth of all participants would not have participated in the study without the possibility of self-organized blood collection in addition to blood collection in the research institute. Nearly all collected blood sample could be analyzed in the laboratory. Further studies could implement self-organized blood collection when investigating serology but should include the characteristics of the study participants to make the decision.

## Supporting information

S1 File (DOCX)

S1 Data (SAV)
