# Supplementary material for: Feasibility of self-organized blood sample collection in adults for study purposes in a primary care setting
Source: PLoS One. 2023 May 25;18(5):e0286014. doi: 10.1371/journal.pone.0286014 (PMC10212115; doi:10.1371/journal.pone.0286014)
Supplement: S1 File — (DOCX) [file pone.0286014.s001.docx]

Supplemental Material

**Figure S1**. Capillary blood kit given to all study participants in a prepaid shipping box


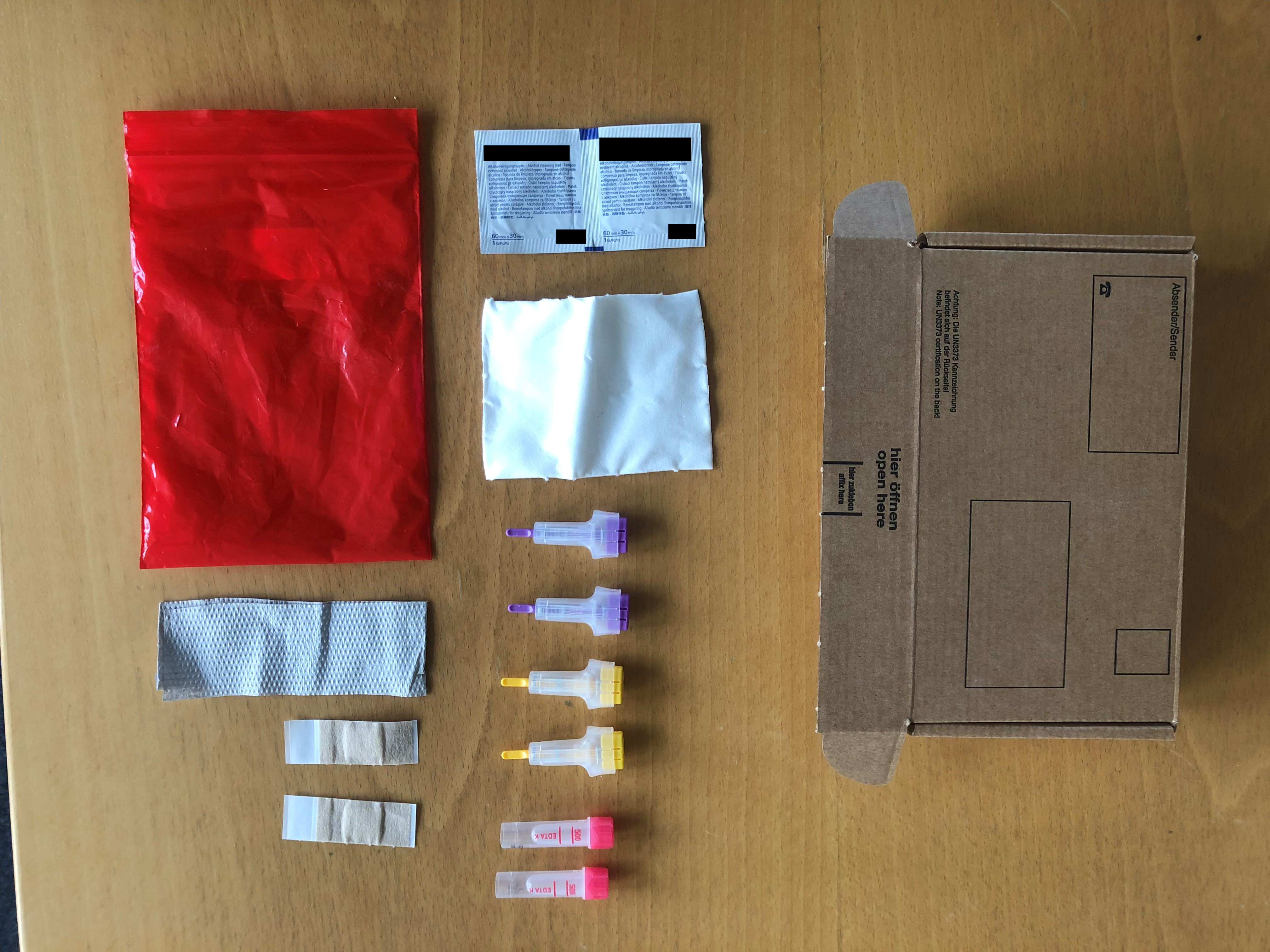


Table S1. Participants not reached during telephone follow-up or opt-out for recontacting (N=269)

| Sex | n (%) |
| --- | --- |
| Male | 93 (34.6) |
| Female | 125 (46.5) |
| Missing | 51 (19.0) |
| Age | |
| Mean (standard deviation) | 62.72 (18.11) |
| < 40 years | 24 (10.3) |
| 40 – 65 years | 109 (40.5) |
| > 65 years | 99 (36.8) |
| Missing | 37 (13.8) |
| High risk group* | |
| 80+ years old | 69 (25.7) |
| Immunosuppressed | 53 (19.7) |
| Haematological or oncological disease | 84 (31.2) |
| Missing | 86 (32.0) |
| School education | |
| Low | 55 (20.1) |
| Middle | 59 (21.9) |
| High | 93 (34.6) |
| Not specified | 6 (2.2) |
| Missing | 56 (20.8) |
| City resident size | |
| < 5,000 | 71 (26.4) |
| 5,000 – 20,000 | 33 (12.3) |
| 20,000 – 100,000 | 34 (12.6) |
| > 100,000 | 62 (23.0) |
| Missing | 69 (25.7) |
| Nursing or medical profession | |
| yes | 26 (9.7) |
| Missing | 45 (16.7) |
| Co-Morbidities* | |
| Hypertension | 93 (34.6) |
| Heart failure | 10 (3.7) |
| Diabetes type 1 | 8 (3.0) |
| Diabetes type 2 | 16 (5.9) |
| Chronic obstructive pulmonary disease | 10 (3.7) |
| Missing | 45 (16.7) |

Data is n (%) if not otherwise stated; *multiple selection possible
